# Factors Associated with Increased Risk of Unplanned Hospital Readmission after Endovascular Aortoiliac Interventions

**DOI:** 10.7759/cureus.3558

**Published:** 2018-11-07

**Authors:** Pardis Pooshpas, Erik Lehman, Faisal Aziz

**Affiliations:** 1 Miscellaneous, Penn State College of Medicine, Penn State Milton S. Hershey Medical Center, Hershey, USA; 2 Surgery, Penn State College of Medicine, Penn State Milton S. Hershey Medical Center, Hershey, USA; 3 Cardiac/thoracic/vascular Surgery, Penn State College of Medicine, Penn State Milton S. Hershey Medical Center, Hershey, USA

**Keywords:** 30 day readmission, vascular surgery, endovascular intervention

## Abstract

Objectives

Readmissions to hospital after surgical procedures are considered as reflective of poor quality of healthcare provided during the index hospitalization and are associated with increased costs of healthcare. Aortoiliac occlusive disease represents an aggressive form of atherosclerotic disease and has been traditionally treated with open surgical bypasses. Endovascular interventions for aortoiliac occlusive disease are associated with comparable outcomes to open surgical procedures. The purpose of this study is to review the factors associated with hospital readmission after aortoiliac endovascular interventions.

Methods

The 2015 procedure targeted American College of Surgeons National Surgical Quality Improvement Program (NSQIP) database and general and vascular surgery NSQIP participant user file (PUF) were used for this analysis. Patient, diagnosis and procedure characteristics of patients undergoing aortoiliac endovascular interventions were reviewed. Bivariate analysis was used to identify the relationship between the independent variables and 30-day readmission. The significant variables from the bivariate analysis were used to generate a multivariable logistic regression model. The predicted probability of readmission was calculated.

Results

Out of 823 patients, 86 were readmitted. Readmission was related to the principal procedure in 48 (73.9%) patients. A total of 61 (7%) patients underwent an unplanned operation within 30 days after the index procedure. A multivariable logistic regression model identified the following variables to be significantly associated with 30-day risk of readmission: the use of pre-procedural beta blocker (OR = 2.06, 95% CI = 1.23 - 3.45, P < 0.01), external/internal iliac intervention (OR = 1.95, 95% CI = 1.18 - 3.20, P <0.01), critical limb ischemia (OR = 1.80, 95% CI = 1.10 - 2.94, P <0.05), and unplanned return to the operating room (OR = 11.65, 95% CI = 6.35 - 21.35, P <0.01). The predicted probability of readmission was as follows: 5.5% for critical limb ischemia, 5.9% for external iliac artery angioplasty/stenting, 6.2% for preoperative beta blockers, 17.7% for patients with cardiac arrest, 27% for unplanned return to the operating room, and 94.7% for patients with all of these risk factors.

Conclusion

Readmissions after endovascular interventions for severe atherosclerotic disease can be used as a quality metric. Several factors place a patient at a high risk for readmission. Unplanned return to the operating room, cardiac arrest, preoperative beta blockers, location of disease, and preoperative symptoms are independent risk factors for hospital readmission. Unplanned return to the operating room is associated with 11.65-fold increase in the risk of hospital readmission.

## Introduction

Readmissions to hospital after surgical procedures are being increasingly used as a quality metric to reflect on the healthcare provided during the index hospitalization. They are generally considered preventable and are associated with significant financial losses for the institutions. Section 3025 of Patient Protection and Affordable Care Act now holds hospitals responsible for early readmissions [1]. Introduction of payment for performance models has increased the scrutiny of individual providers and gives the institutions an opportunity to incentivize healthcare providers who meet the quality metrics. Administrative databases are being extensively used to determine such occurrences. The American College of Surgeons National Surgical Quality Improvement Program (ACS-NSQIP) started capturing surgical readmission data in 2011 and numerous studies have used this database to identify the factors associated with readmission after surgical procedures [2-4]. This issue is of particular importance for vascular surgeons, as vascular operations follow congestive heart failure and psychosis as the third most common reason for hospital readmission. The same study showed that among the surgical specialties, vascular surgery was associated with the highest incidence of postoperative hospital admissions [5].

Peripheral arterial disease (PAD) represents a significant form of systemic atherosclerotic disease and patients with PAD usually have multiple, serious medical conditions that may be responsible for perioperative complications. PAD is one of the most common conditions treated by vascular surgeons. The anatomic level for PAD can be divided into two groups: aortoiliac occlusive disease and infrainguinal occlusive disease. Aortoiliac occlusive disease is different from infrainguinal disease in multiple aspects: it represents an aggressive form of atherosclerosis; patients often present with severe, disabling claudication or critical limb ischemia, and the long-term outcomes of endovascular treatment of aortoiliac occlusive disease are approximately comparable to open surgical operations to treat this arterial segment. Hence, it is an attractive option for vascular surgeons to offer endovascular therapy to such patients. Incidence of readmission after lower extremity revascularization varies from 15% to 26% [6, 7]. Factors associated with hospital readmission after endovascular interventions [4] or surgical bypasses [2] for patients with infrainguinal disease have been previously described. The purpose of this study is to analyze the factors associated with unplanned readmissions after endovascular treatment of the aortoiliac occlusive disease. Since previously published data based on ACS-NSQIP has been shown to be reliable and reproducible in multiple studies, the authors chose this database for this analysis.

## Materials and methods

Data file

The American College of Surgeons National Surgical Quality Improvement Program (ACS-NSQIP) collects de-identified data from more than 300 participating institutions across the country. In order to make it compliant with Health Insurance Portability and Accountability Act (HIPAA), all data is de-identified. The ACS provides this data in the form of Participant Use Data Files (PUF) [8]. Since the data is de-identified, there is no requirement to obtain approval from an institutional review board. A clinical nurse, trained specifically for NSQIP data entry at each participating site, collects more than 150 preoperative, intraoperative, and postoperative variables for surgical procedures. A systemic sampling method is used that gathers data from the first 40 operations performed within each eight-day cycle while applying certain case exclusion and hospital exclusion criteria. The data collected by the ACS NSQIP is used for research studies and quality assessment and improvement purposes. Methods for data extraction from this database have been described previously [9-11] and the outcomes of these data files are reproducible and reliable with audits showing less than 2% disagreement rates [12].

Patients

All patients who underwent endovascular aortoiliac intervention during the calendar year 2015 were identified using Procedure-Targeted-PUF. This file was then merged with the main ACS NSQIP PUF file using unique case identification numbers.

Outcomes

The primary outcome was readmission within 30 days after the procedure. Patients were divided into two groups:  'readmission within 30 days' and 'no readmission'. Several preoperative variables were included in this analysis: age, sex, days from admission to operation, American Society of Anesthesiology (ASA) classification, physiologic risk factors, smoking status, chronic obstructive pulmonary disease (COPD), congestive heart failure (CHF), pneumonia, hypertension (HTN), use of antiplatelet medications, use of beta blockers, use of statins, diabetes mellitus (DM), dialysis dependence, indications for surgery, and transfer from another hospital. The intraoperative variables included in this analysis were operative time, high risk anatomical factors, type of operation, and emergency operation. The postoperative variables included length of hospital stay, bleeding requiring transfusions, myocardial infarction, stroke, major re-intervention of treated arterial segment, untreated loss of patency, wound infection, unplanned intubation, renal insufficiency, acute renal failure, urinary tract infection, stroke, cardiac arrest, and unplanned return to the operating room.

Statistical analysis

All analyses were performed using SAS version 9.4 (SAS Institute, Cary, NC), and statistical significance was set at 0.05. Prior to analysis, all variables were summarized with frequencies and percentages or with means, medians, and standard deviations. The distributions of continuous variables were assessed using histograms and normal probability plots. Potential predictors of 30-day readmission were tested using logistic regression in a bivariate analysis. Exact logistic regression was substituted as needed based on model assumptions, and odds ratios were used to quantify the magnitude and direction of any significant associations. The significant (p<0.05) predictors from the bivariate analysis were then included in a process of stepwise selection to find the group of variables that collectively were most significantly associated with 30-day readmission. Prior to selection, this group of variables was tested for multicollinearity using variance inflation factor (VIF) statistics. Forward and backward selection was used as a check for other potential models, but all approaches resulted in the same model. Finally, the fit of the final model was assessed using the Hosmer-Lemeshow goodness-of-fit test (p=0.89). A prediction equation for the probability of 30-day readmission was constructed using the parameter estimates from the final model, and predicted probabilities for certain characteristics and combinations of characteristics were calculated using this equation.

## Results

Demographics and preoperative comorbidities

**Figure 1:**
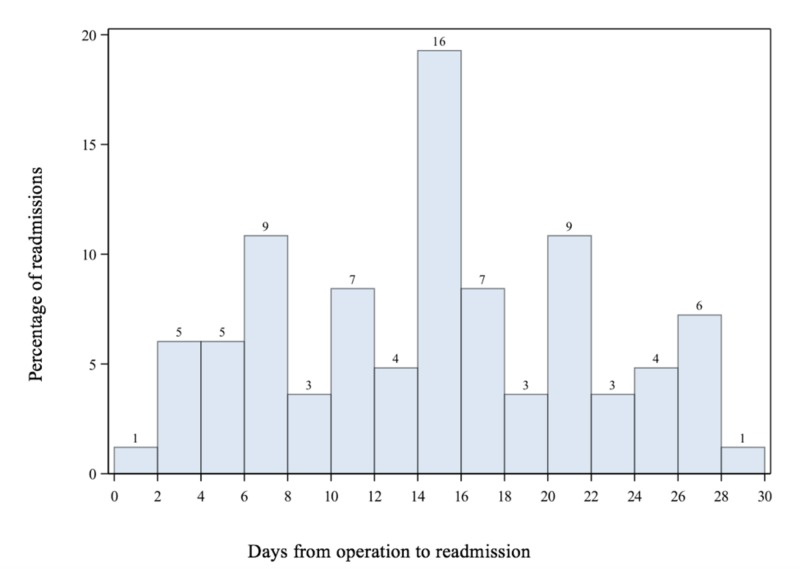
30-day Readmission Percentage of patients with 30-day readmission vs. days from operation to readmission. The mean for number of days from procedure to readmission was 14.22 ± 7.29.

A total of 823 patients (42% female, 58% males) underwent endovascular aortoiliac interventions in the calendar year 2015. The mean age was 64.60 (±10.83) years. The distribution of race was as follows: White (84.14%), African American (15.07%), Asian (0.31%), Native Hawaiian (0.31%), and American Indian (0.16%). Of total 823 patients, 86 (10.5%) patients were readmitted to hospital within 30 days after the index procedure. The mean for number of days from procedure to readmission was 14.22 ± 7.29 with 60% of the readmissions occurring within 16 days after the procedure (Figure 1).

Comparing variables in terms of readmissions 

Through a bivariate analysis using logistic regression, the following variables were found to have no significant association with these two groups: age, age range, sex, time from admission to operation, ASA classification, presence of high physiological risk factors, current smoking status, history of COPD, congestive heart failure, HTN, preoperative use of statins, dialysis dependence, transfer from another institution, operation time, operation time range, presence of anatomic high risk factors, emergency operation, length of hospital stay, unplanned re-intubation, renal insufficiency, urinary tract infection, and cerebrovascular accident (CVA)/stroke with neurologic deficit.

Using this same analysis, the following factors were found to be associated with significantly high risk of 30-day readmission after surgery: use of preoperative antiplatelet medications (OR 2.78, CI 1.26-6.14, p=0.01), preoperative use of beta blockers (OR 1.85, CI 1.16-2.95, p=0.01), insulin-dependent diabetes mellitus (OR 2.11, CI 1.21-3.66, p=0.03), presence of critical limb ischemia (OR 2.21, CI 1.41-3.47, p<0.01), external iliac artery angioplasty and/or stenting (OR 2.05, CI 1.30-3.23, p<0.01), postoperative bleeding requiring transfusion or secondary procedure (OR 4.58, CI 2.32-9.04, p<0.01), combined outcome of MI or stroke (OR 5.10, CI 1.46-17.76, p=0.01), MI (OR 4.40, CI 1.08-17.94, p=0.04), major re-intervention of treated arterial segment (OR 7.35, CI 3.54-15.27, p<0.01), untreated loss of patency (OR 8.84, CI 1.76-44.52, p=0.01), wound infection (OR 2.10, CI 1.22-3.62, p=0.01), cardiac arrest requiring cardiopulmonary resuscitation (CPR) (OR 5.29, CI 1.24-22.54, p=0.02), and unplanned return to the operating room (OR 11.21, CI 6.34-19.83, p<0.01) (Table 1).

**Table 1:** Variables Associated with 30-day Readmission After Endovascular Aortoiliac Interventions ASA, American Society of Anesthesiologists; COPD, Chronic obstructive pulmonary disease; NIDDM, non-insulin-dependent diabetes mellitus; IDDM, insulin-dependent diabetes mellitus; MI, myocardial infarction; CVA, cerebrovascular accident; CPR, cardiopulmonary resuscitation; OR, odds ratio; CI, confidence interval. Continuous variables presented as mean ± standard deviation; categorical variables presented as number (%). Bold values belong to statistically significant factors.

	No Readmission	Readmission	OR	P value
	(N = 737)	(N = 86)	(95% CI)	
Preoperative Variables				
Age (years)	64.53 ± 10.95	65.23 ± 9.71	1.03 (0.93, 1.14)	0.57
Age range (years)				0.54
- <60	238 (90.8)	24 (9.2)	Reference	
- 60-69	257 (88.9)	32 (11.1)	1.24 (0.71, 2.16)	
- 70-79	168 (87.5)	24 (12.5)	1.42 (0.78, 2.58)	
- ≥ 80	74 (92.5)	6 (7.5)	0.80 (0.32, 2.04)	
Sex				0.59
- Male	423 (89.1)	52 (11.0)	Reference	
- Female	314 (90.2)	34 (9.8)	0.88 (0.56, 1.39)	
Time from Admission to operation (days)	0.65 ± 2.76	1.01 ± 2.63	1.02 (0.99, 1.05)	0.29
ASA classification				0.64
- No/Mild Disturb	61 (89.7)	7 (10.3)	Reference	
- Severe Disturb	410 (90.5)	43 (9.5)	0.91 (0.39, 2.12)	
- Life Threat/Moribund	161 (88.0)	22 (12.0)	1.19 (0.48, 2.93)	
High physiologic risk factors				0.15
- No	637 (90.4)	68 (9.7)	Reference	
- Yes	90 (85.7)	15 (14.3)	1.56 (0.86, 2.85)	
Current smoker within one year				0.53
- No	352 (90.3)	38 (9.7)	Reference	
- Yes	385 (88.9)	48 (11.1)	1.16 (0.74, 1.81)	
History of COPD				0.47
- No	630 (89.9)	71 (10.1)	Reference	
- Yes	107 (87.7)	15 (12.3)	1.24 (0.69, 2.25)	
Congestive heart failure in 30 days before surgery				0.07
- No	720 (89.9)	81 (10.1)	Reference	
- Yes	17 (77.3)	5 (22.7)	2.62 (0.94, 7.28)	
Hypertension requiring medication				0.13
- No	193 (92.3)	16 (7.7)	Reference	
- Yes	544 (88.6)	70 (11.4)	1.55 (0.88, 2.74)	
Preoperative antiplatelet				0.01
- No	145 (95.4)	7 (4.6)	Reference	
- Yes	589 (88.2)	79 (11.8)	2.78 (1.26, 6.14)	
Preoperational beta blocker				0.01
- No	365 (92.4)	30 (7.6)	Reference	
- Yes	368 (86.8)	56 (13.2)	1.85 (1.16, 2.95)	
Preoperational statin				0.07
- No	223 (92.5)	18 (7.5)	Reference	
- Yes	511 (88.3)	68 (11.7)	1.65 (0.96, 2.84)	
Diabetes				0.03
- No	506 (91.2)	49 (8.8)	Reference	
- NIDDM	128(88.9)	16 (11.1)	1.29 (0.71, 2.34)	
- IDDM	103(83.1)	21 (16.9)	2.11 (1.21, 3.66)	
Currently on dialysis				0.13
- No	711 (89.9)	80 (10.1)	Reference	
- Yes	26 (81.3)	6 (18.8)	2.05 (0.82, 5.13)	
Symptomatology				<0.01
- Asymptomatic/Claudication	467 (92.3)	39 (7.7)	Reference	
- Critical limb ischemia: rest pain/tissue loss	255 (84.4)	47 (15.6)	2.21 (1.41, 3.47)	
Transferred				0.87
- No	55 (90.2)	6 (9.8)	Reference	
- Yes	681(89.5)	80 (10.5)	0.93 (0.39, 2.23)	
Intraoperative Variables				
Operation time	104. 31 ± 75.52	116 ± 104.05	1.05 (0.98, 1.14)	0.18
Operation time range				0.18
- < 1 hr	245 (89.4)	29 (10.6)	Reference	
- 1-2 hrs	269 (89.4)	32 (10.6)	1.01 (0.59, 1.71)	
- 2-3 hrs	116 (94.3)	7 (5.7)	0.51 (0.22, 1.20)	
- ≥ 3 hrs	107 (85.6)	18 (14.4)	1.42 (0.76, 2.67)	
High risk anatomic factors				0.09
- None/Not documented	506 (91.2)	49 (8.8)	Reference	
- Prior ipsilateral bypass involving currently treated segment	102 (86.4)	16 (13.6)	1.62 (0.89, 2.96)	
- Prior ipsilateral percutaneous intervention involving currently treated segment	129 (86.0)	21 (14.0)	1.68 (0.97, 2.90)	
Location of Disease				<0.01
- Common Iliac stenting	534 (91.6)	49 (8.4)	Reference	
- External/internal iliac angioplasty/stenting	197 (84.2)	37 (15.8)	2.05 (1.30, 3.23)	
Emergency operation				0.41
- No	706 (89.4)	84 (10.6)	Reference	
- Yes	31 (94.0)	2 (6.1)	0.54 (0.13, 2.31)	
Postoperative Variables				
Length of total hospital stay (days)	2.81 ± 6.32	3.05 ± 4.28	1.01 (0.97, 1.04)	0.73
Length of total hospital stay (days)				0.06
- 0	287 (90.8)	29 (9.2)	0.62 (0.37, 1.04)	
- 1	217 (92.0)	19 (8.1)	0.54 (0.30, 0.96)	
- 2+	233 (86.0)	38 (14.0)	Reference	
Bleeding requiring transfusion or secondary procedure				<0.01
- No	707 (90.8)	72 (9.2)	Reference	
- Yes	30 (68.2)	14 (31.8)	4.58 (2.32, 9.04)	
MI or stroke				0.01
- No	730 (89.9)	82 (10.1)	Reference	
- Yes	7 (63.6)	4 (36.4)	5.10 (1.46, 17.76)	
MI				0.04
- No	731 (89.8)	83 (66.7)	Reference	
- Yes	6 (66.7)	3 (33.3)	4.40 (1.08, 17.94)	
Major reintervention of treated arterial segment				<0.01
- No	718 (90.9)	72 (9.1)	Reference	
- Yes	19 (57.6)	14 (42.4)	7.35 (3.54, 15.27)	
Untreated loss of patency				0.01
- No	734 (89.8)	83 (10.2)	Reference	
- Yes	3 (50.0)	3 (50.0)	8.84 (1.76, 44.52)	
Wound infection				0.01
- No	644 (90.7)	66 (9.3)	Reference	
- Yes	93 (82.3)	20 (17.7)	2.10 (1.22, 3.62)	
Unplanned re-intubation				0.41
- No	728 (89.7)	84 (10.3)	Reference	
- Yes	9 (81.8)	2 (18.2)	1.93 (0.41, 9.06)	
Renal insufficiency				0.13
- No	736 (89.7)	85 (10.4)	Reference	
- Yes	1 (50.0)	1 (50.0)	8.66 (0.54, 139.70)	
Urinary tract infection				0.09
- No	4 (66.7)	2 (33.3)	Reference	
- Yes	733 (89.7)	84 (10.3)	4.36 (0.79, 24.18)	
CVA/stroke with neurological deficit				0.13
- No	736 (89.7)	85 (10.4)	Reference	
- Yes	1 (50.0)	1 (50.0)	8.66 (0.54, 139.70)	
Cardiac arrest requiring CPR				0.02
- No	5 (62.5)	3 (37.5)	Reference	
- Yes	732 (89.8)	83 (10.2)	5.29 (1.24, 22.54)	
Unplanned return to the operating room				<0.01
- No	705 (92.5)	57 (7.5)	Reference	
- Yes	32 (52.5)	29 (47.5)	11.21 (6.34, 19.83)	

Multivariable analysis

Multivariable analysis identified the following factors to be associated with significant risk of readmission: unplanned return to the operating room (OR 11.65, 95% CI 6.35 - 21.35, P <0.01), cardiac arrest requiring CPR (OR 6.70, 95% CI 1.42 - 31.73, P = 0.02), preoperative use of beta blocker (OR 2.06, 95% CI 1.23 - 3.45, P < 0.01), external/internal iliac angioplasty/stenting vs. common Iliac stenting (OR 1.95, 95% CI 1.18 - 3.20, P <0.01), and critical limb ischemia (OR 1.80, 95% CI 1.10 - 2.94, P = 0.02) (Table 2).

**Table 2:** Multivariable Logistic Regression Model OR, odds ratio; CI, confidence interval.

Risk Factor	Adjusted OR (95% CI)	P value
Preoperational beta blocker	2.06 (1.23, 3.45)	< 0.01
Location of Disease (External Iliac artery)	1.95 (1.18, 3.20)	< 0.01
Symptomatology (Critical Limb Ischemia)	1.80 (1.10, 2.94)	0.02
Cardiac arrest requiring CPR	6.70 (1.42, 31.73)	0.02
Unplanned return to the operating room	11.65 (6.35, 21.35)	< 0.01

Most severe procedural outcomes

The most severe procedural complications were as follows: death (n=8, 1.7%), major amputation (n=7, 1.4%), new bypass in the treated segment (n=14, 2.9%), and recurrent stenosis (n=33, 6.8%).

Reasons for readmission

As per the judgment of the ACS-NSQIP coordinators at the respective institutions, out of the 65 unplanned readmissions, 48 (74%) were deemed related to the principal procedure of aortoiliac endovascular intervention. Of this subgroup of patients, the reasons for readmission included bleeding requiring transfusion, deep surgical site infection, myocardial infarction, pneumonia, pulmonary embolism, septic shock, superficial surgical site infection, urinary tract infection, deep venous thrombosis requiring therapy, and unspecified reasons (Table 3).

**Table 3:** Reasons for Readmission SSI, surgical site incision.

Occurrences	Number (%)
Total Number of Unplanned Readmissions	65
- Related to Principal Procedure	48 (74%)
- Unrelated to Principal Procedure	17 (26%)
Reasons for Readmissions Deemed Related to the Principal Procedure (N = 48)	
Bleeding Requiring Transfusion	1/48 (2.1)
Deep Incisional SSI	3/48 (6.3%)
Myocardial Infarction	1/48 (2.1%)
Pneumonia	2/48 (4.2%)
Pulmonary Embolism	1/48 (2.1%)
Septic Shock	2/48 (4.1%)
Superficial Incisional SSI	2/48 (4.1%)
Urinary Tract Infection	1/48 (2.1%)
Deep Vein Thrombosis Requiring Therapy	1/48 (2.1%)

Predicted probability of readmission

The predicted probability of readmission was calculated for risk factors obtained from the multivariable model. The predicted probability of readmission was 5.5% for patients with critical limb ischemia, 5.9% for patients undergoing external iliac artery angioplasty/stenting, 6.2% for patients with pre-procedural beta blocker use, 17.7% for patients suffering from postoperational cardiac arrest requiring CPR, and 27% for patients who returned to the operating room. In the presence of all the factors from the multivariable model, the predicted probability of readmission was 94.7%. In the absence of all the factors, the predicted probability of readmission was 3.1% (Table 4).

**Table 4:** Predicted Probability of Readmission CPR, cardiopulmonary resuscitation.

Critical Limb Ischemia	External Iliac Angioplasty/Stenting	Preoperative Use of Beta Blockers	Cardiac Arrest Requiring CPR	Unplanned return to the operating room	Probability of Readmission (%)
–	–	–	–	–	3.10
+	–	–	–	–	5.50
–	+	–	–	–	5.90
–	–	+	–	–	6.20
–	–	–	+	–	17.70
–	–	–	–	+	27
+	+	+	+	+	94.70

## Discussion

Aortoiliac occlusive disease represents an advanced form of atherosclerotic vascular disease. Patients with aortoiliac occlusive disease generally have multilevel disease and can present with life-limiting claudication or critical limb ischemia. The goal for revascularization in such patients is to establish inline flow to the lower extremities. Open surgical revascularization (aortobifemoral bypass) is the gold standard surgical operation for effective treatment of aortoiliac occlusive disease; however, with advances in endovascular technology, more aortoiliac atherosclerotic lesions are treated with endovascular interventions. Trans-Atlantic Inter-Society Consensus (TASC) II guidelines are the most widely accepted guidelines for the treatment of aortoiliac occlusive disease. These guidelines are solely based on the morphology of the lesion and don’t take the patients' physiologic risk factors into account. The current guidelines recommend endovascular therapy for less complex lesions and open surgical revascularization for patients with TASC-C and D lesions [13]. Unfortunately, the guidelines don’t take the patients’ extensive comorbidities and the perioperative risks and complications of aortobifemoral bypass into consideration. Surgical revascularization procedures for aortoiliac occlusive disease are complicated operations, associated with significantly high risk of perioperative morbidity and mortality [14-16]. The majority of the patients with aortoiliac occlusive disease have a combination of serious systemic comorbidities, which place them at a substantially high physiologic risk for undergoing major vascular surgery procedures. While aortobifemoral bypass is an ideal operation for a patient with low risk of developing perioperative complications, it is a high-risk operation for a substantial number of patients with aortoiliac occlusive disease. Over time, the techniques of endovascular operations are getting more refined and modern-day vascular surgeons are more well-equipped to treat morphologically complicated lesions with endovascular techniques. 

Minimally invasive interventions are associated with significantly lower risk of perioperative morbidity and mortality [17]. The only randomized controlled trial published to date, comparing endovascular treatment to open operations for severe infrainguinal arterial disease clearly shows that patients with life expectancy more than two years benefit from surgical revascularization [18]. However, this trial does not include patients with aortoiliac occlusive disease. Recent literature shows comparable patency rates between surgical bypasses and endovascular interventions to treat aortoiliac occlusive disease [19-21]. It is not surprising that during the time period between 1996 and 2000, stenting and angioplasty for aortoiliac occlusive disease increased by 850% with a concurrent 16% decrease in the incidence of open surgery to treat aortoiliac occlusive disease [17]. It most likely represents a combination of increasing comfort level of vascular surgeons to treat aortoiliac occlusive disease with endovascular modalities and high incidence of perioperative morbidity and mortality associated with aortobifemoral bypass when compared to endovascular interventions.

This analysis has several important findings. The incidence of readmission after endovascular treatment of aortoiliac occlusive disease is 10.5%. Since the endovascular procedures are inherently minimally invasive, this number can be considered significant. Unlike readmissions after index hospitalization for a medical admission, early readmission to a surgical service is generally due to postoperative complications [22]. A recent review of the Medicare claims database shows that the cost of hospital readmissions can add up to 12 billion dollars a year [5]. A recent analysis by Lawson et al. [23] has shown that reducing the postoperative complications by 5% for the 20 procedures associated with the highest readmission rates can prevent over 2000 readmissions and save Medicare close to 31 million dollars yearly. In addition, if all the complications were mitigated, a total of 41,846 readmissions can be prevented and over 600 million dollars could be saved. Our data also shows that among 74% of the readmitted patients, reason for readmission was deemed to be related to the principal procedure. The multivariable analysis identified the following factors to be associated with increased risk of hospital readmission after endovascular aortoiliac intervention: unplanned return to the operating room (OR 11.65), postoperative cardiac arrest (OR 6.7), use of preoperative beta blockers (OR 2.06), anatomic location of disease being external iliac artery (OR 1.95), and presence of critical limb ischemia (OR 1.8). Unplanned return to the operating room is generally considered a preventable event. Table 3 highlights the reasons for readmissions after aortoiliac endovascular interventions. Just like other surgical procedures, most of the readmissions (74%) are deemed to be related to the principal procedure. Unfortunately, among this cohort of patients, reasons for readmission of majority (71%) of patients were not reported. Missing crucial information from large surgical databases could in part be attributed to non-uniformity in use of current procedural terminology codes (CPT) across different institutions. With the use of the International Classification of Disease -10 (ICD-10) system, there will be enhanced specificity in medical documentation.

The ACS-NSQIP database does not provide intraoperative findings discovered during reoperation. This reflects an area needing improvement in future versions of this database. Review of general surgery literature [24-25] shows that unplanned return to the operating room is likely attributed to multifactorial etiology and technical mistakes while errors in clinical judgment are likely responsible for majority of such events. In general, common complications of endovascular interventions that require reoperation are related to bleeding or thrombosis. Close attention to technical details to ensure adequate access, details of endovascular intervention, appropriate therapy (balloon angioplasty, stenting, etc.) of the lesion, and control of contrast extravasation or thrombosis are the key elements of successful endovascular procedures. While bleeding from infrainguinal blood vessels can be dangerous, it is usually self-contained. Bleeding from iliac blood vessels, on the other hand, can be fatal due to the anatomic location of iliac arteries and the fact that retroperitoneum can accommodate large volume of blood leading to rapid development of hemorrhagic shock. Hence, endovascular interventions for aortoiliac occlusive disease should be performed with great caution utilizing the basic principles of endovascular surgery, as patients with bleeding complications from iliac vessels usually deteriorate rapidly. Endovascular interventionists performing such procedures should be familiar with and be ready to use techniques of balloon occlusion and stent graft coverage of active areas of hemorrhage from the iliac arteries, if such a situation arises.

Hospital readmissions reflect poorly on the quality of healthcare provided and are associated with significant costs. The resources that are employed to take care of readmitted patients could be used to provide care to new patients. Reducing readmissions can increase the number of beds available in hospitals and may reduce the emergency room wait times. With increasing scrutiny from governmental agencies and implementations of payment for performance models, there has been an increasing focus on identifying the risk factors associated with hospital readmissions with the goal of reducing such events. Prevention of readmissions not only enhances the quality of healthcare and improves efficiency of the healthcare systems, but it also reduces healthcare costs. Patients with PAD generally have several serious comorbidities that place them at a high risk for developing perioperative complications. This analysis shows that the patients presenting with critical limb ischemia, diabetes, and using preoperative beta blockers are at a high risk for needing readmissions. These results imply that the incidence of readmission is higher among patients with serious comorbidities. In addition, the risk of readmission was higher for patients who received endovascular treatment of the external iliac arteries. Another study has shown that patients with external iliac artery stents had more extensive lesions, poorer run-offs, smaller vessel size and significantly reduced primary patency rates as compared to patients who received stenting for common iliac arteries [26]. Despite these challenges, some factors can still be mitigated or controlled to reduce the incidence of complications. This analysis shows that for patients who require unplanned return to the operating room, the incidence of hospital readmission is 27% and for patients who have all of the five risk factors (unplanned return to the operating room, cardiac arrest, preoperative use of beta blockers, external iliac artery intervention, and presence of critical limb ischemia), the risk of readmission can be as high as 95%. These results have important implications, as they identify the risk factors associated with high incidence of hospital readmission. While most of these factors may be considered non-modifiable, unplanned return to the operating room could be preventable and every effort should be made to avoid such occurrences. These incidences reflect opportunities to improve quality of care while reducing medical waste and overall medical costs.

This study has several limitations. It is a retrospective analysis that comes with all the pitfalls of any retrospective study. The ACS-NSQIP data is self-reported by the institutions. The variables are limited to those provided by the ACS-NSQIP. The findings encountered during the reoperation are not recorded in the database. The outcomes are limited to 30 days after operation and hence, any readmissions after this time period are not recorded in this dataset. The advantages of this study are that ACS-NSQIP is the largest surgical database available to surgeons in the US and previous publications have shown that results based on this database are highly reliable and reproducible. Being a national database, it includes all surgeons across the country, both from academic institutions and from community hospitals. Given the large number of participating hospitals in this data, it provides us with an accurate, bigger picture of aortoiliac interventions performed across the US and associated complications, including hospital readmissions.

## Conclusions

An increasing number of organizations are recognizing unplanned readmissions after elective operations as indicators for poor quality of healthcare provided during index hospitalizations. We can only predict that over time, such occurrences will be scrutinized more heavily by healthcare agencies. Aortoiliac occlusive disease represents an aggressive form of atherosclerotic disease, which is increasingly being treated with endovascular interventions with excellent long-term outcomes. Unplanned return to the operating room after endovascular interventions for aortoiliac occlusive disease is associated with 11.65-fold risk of hospital readmission. Every effort should be made to avoid technical errors during the operation to avoid postoperative complications. The decision to bring a patient back to the operating room requires sound clinical judgment and should be made after careful calculation of the risk to benefit ratio.
